# Hyperactive mariner transposons are created by mutations that disrupt allosterism and increase the rate of transposon end synapsis

**DOI:** 10.1093/nar/gkt1218

**Published:** 2013-12-05

**Authors:** Danxu Liu, Ronald Chalmers

**Affiliations:** School of Life Sciences, University of Nottingham, Queen’s Medical Centre, Nottingham NG7 2UH, UK

## Abstract

New applications for transposons in vertebrate genetics have spurred efforts to develop hyperactive variants. Typically, a genetic screen is used to identify several hyperactive point mutations, which are then incorporated in a single transposase gene. However, the mechanisms responsible for the increased activity are unknown. Here we show that several point mutations in the mariner transposase increase their activities by disrupting the allostery that normally serves to downregulate transposition by slowing synapsis of the transposon ends. We focused on the conserved WVPHEL amino acid motif, which forms part of the mariner transposase dimer interface. We generated almost all possible single substitutions of the W, V, E and L residues and found that the majority are hyperactive. Biochemical analysis of the mutations revealed that they disrupt signals that pass between opposite sides of the developing transpososome in response to transposon end binding. In addition to their role in allostery, the signals control the initiation of catalysis, thereby preventing non-productive double-strand breaks. Finally, we note that such breaks may explain the puzzling ‘self-inflicted wounds’ at the ends of the Mos1 transposon in *Drosophila*.

## INTRODUCTION

For many years, the Tn*10* and P element transposons have been used successfully as genetic tools in *Escherichia coli* and *Drosophila* (1,2). More recently, Sleeping Beauty and PiggyBac systems have been developed for cancer gene discovery and transgenic applications in vertebrates (3–5). By combining several independently isolated point mutations, the PiggyBac and Sleeping Beauty transposition rates have been increased by 17- and 100-fold, respectively (6,7).

For most genes, such as those involved with housekeeping functions, it is difficult to generate hyperactive variants. This is because biological systems usually occupy summits on the local fitness landscape. In contrast, it is relatively easy to isolate hyperactive eukaryotic transposases. The explanation may be that transposons that have acquired detrimental mutations continue to be amplified owing to the active transposase provided by other copies of the element. Therefore, this relaxation of natural selection may allow the transposases sequences to drift off the local fitness summit (8,9). Furthermore, transposons are probably also subject to stabilizing selection, which will balance their selfish amplification against detrimental effects on host fitness. In principle, any variation that reduces activity can mediate stabilizing selection. However, modeling suggests that transposons require active regulation for survival (10). At the start of a genomic invasion, when the copy number is low, a high rate of transposition is desirable to protect the element from genetic drift. Later, as the copy number increases, a progressively lower rate of transposition (per copy) will protect host fitness. Therefore, Hyperactive variants may arise from the reversal of detrimental mutations or the relaxation of autoregulation.

Currently, the most widely used transposon tool is a hyperactive Tn*5*. It is an effective *in vivo* mutagen and has numerous post-genomic applications such as the delivery of sequence bar codes and deep-sequencing primers. Hyperactivity depends on three point mutations, which inactivate the transposon’s natural autoregulatory mechanisms (11,12). Although two of the mutations were selected from random libraries, M56A was informed by prior knowledge of autoregulation.

Our knowledge of autoregulation in eukaryotic transposons remains sparse. However, recent findings show that in the mariner transposon Hsmar1, it arises from a competition for transposase binding sites at the transposon ends (13). We refer to this as an assembly site occlusion (ASO) model. In Hsmar1, the mechanism is augmented by allosteric interactions between the transposase subunits: The free transposase probably has a 2-fold symmetry because this is the lowest energy state for a homodimer. If so, the DNA binding domains will have the same affinity for transposon ends. However, binding of the first transposon end reduces the affinity of the unoccupied site by orders of magnitude (13,14). We view this as a form of allosterism between the subunits because the status of one dictates the properties of the other. The low affinity of the developing transpososome for the second transposon end reduces the rate of synapsis and increases the competition for binding sites, which imposes effective autoregulation at a low transposase concentration (13).

**Figure 1.**
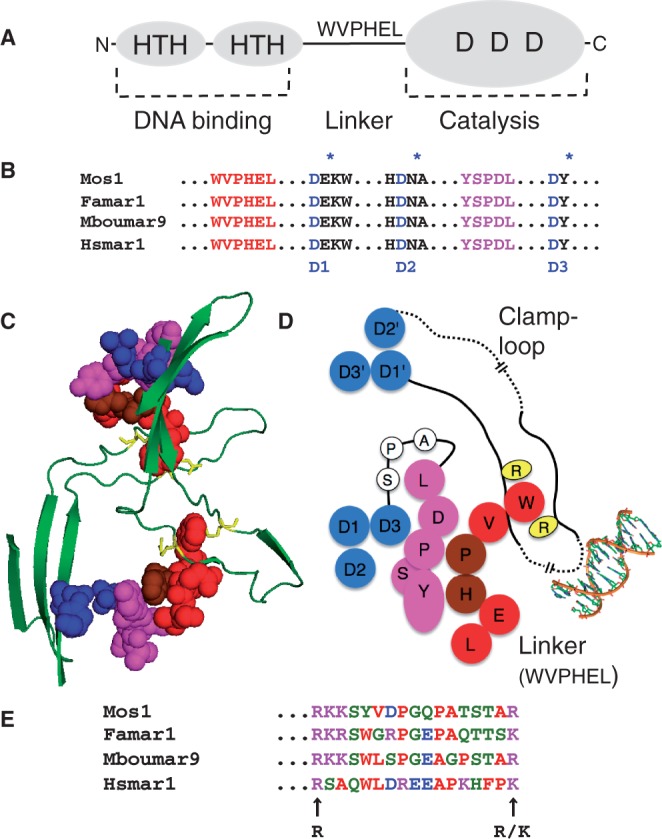
Structural features of the mariner transposase. (**A**) Transposase has an N-terminal DNA binding domain (amino acids 1–115 approximately) with two helix-turn-helix motifs (HTH). The catalytic domain (amino acids 125–343) has a triad of conserved aspartate residues (DDD), which coordinate the catalytic Mg^2+^ ions. The domains are connected by a proteolitically sensitive linker region, which harbors the conserved WVPHEL sequence motif. (**B**) Hsmar1 transposase is aligned with the sequences of three naturally active mariner elements (16–19). In addition to the catalytic triad, mariner has two highly conserved sequence motifs: WVPHEL and YSPDL (20,21). (**C** and **D**) The structural relationships between the conserved sequence motifs as they appear in the crystal structure of the Mos1 post-cleavage intermediate (15). A long unstructured ‘clamp-loop' is found inserted between two strands of the core β sheet. The loop extends across the dimer interface and contacts the WVPHEL motif of the opposite subunit. The tryptophan (W) residue is sandwiched between two arginine residues (R). WVPHEL also contacts the YSPDL sequence motif, which is connected to the third active site D residue by a stretch of three residues (A-P-S). Dashed lines indicate breaks in the drawing scale, which is approximate. Also note that the residues in the two dimensional cartoon are arranged to give the most accurate overall picture of the relationships between the structural elements and that some distortions are inevitable. (**E**) The region of Hsmar1 between the conserved basic residues in the clamp-loop is aligned with the sequences of the three naturally active mariner elements from part B.

The crystal structure of the mariner Mos1 transpososome provides a clear view of the dimer interface in the post-cleavage intermediate. Even though the dimer interface most likely changes as the reaction progresses, the structure highlights regions of the protein that may be determinants of the allosterism. Most of the dimer interface is contributed by the ‘clamp-loop’ and ‘linker’ regions (15). The linker connects the DNA binding and catalytic domains and harbors the conserved WVPHEL motif (Figure 1A and B). The clamp-loop extends from the catalytic core of one monomer and interacts with the WVPHEL motif of the other (Figure 1C and D). The most striking protein–protein contacts are between a pair of conserved arginine (R) residues, which sandwich the plane of the aromatic tryptophan (W) side chain (Figure 1D and E). The clamp-loop also interacts non-specifically with the transposon end on the opposite side of the complex, providing a mechanism whereby binding might be signaled across the complex.

Alanine-scanning mutagenesis of the WVPHEL motif in the related Himar1 yielded hyperactive transposases at four of the six positions (22). In principle, the particular residues could be present owing to genetic drift or stabilizing selection. However, the clustering of the mutations in a conserved motif suggests that their role is active, rather than passive, whereas their location in the heart of the dimer interface suggests that it may involve the aforementioned allostery. In the present work, we characterized almost all possible single mutations of the WVPHEL motif and found that most yield hyperactive transposases. Consistent with location of the mutations in the heart of the dimer interface, we also demonstrated that the hyperactivity arises from the disruption of allostery between the subunits and the resulting relief from autoregulation. Although the overall effect of the mutations is to increase the rate of transposition, the mutations allow off-pathway reactions that damage the DNA.

## MATERIALS AND METHODS

Materials were generally of the best quality available from commercial suppliers. Hsmar1 transposase was expressed, purified and assayed as described in (14,23). Transposase expression plasmids for purification and papillation were created by cloning the respective transposase genes in pMAL c2X (New England Biolabs) between the EcoR1 and BamH1 restriction endonuclease sites. pRC880 encodes the wild-type transposase; pRC1230–1235 encode W118V, W118R, V119T, E122R, L123S, L123N; and pRC1248-1249 encode W118R+E122R and W118R+L123N transposases, respectively. Plasmids pRC1251-1259 encode R166A, K182A, R166A/K182A, W118D/R166A, W118D/K182A, W118D/R166/A/K182A, Y275A, P267A and L278A transposases, respectively. Further details of methods are given in the figure legends. For the papillation assay, expression plasmids were transformed into the reporter strain *E. coli* RC5096 (later in the text). It encodes a transposon with a promoter-less lacZ and a kanamycin resistance gene inserted at a transcriptionally silent chromosomal locus in a lac^−^ strain of *E. coli*. Because the lacZ gene is not expressed, the strain produces white colonies on X-gal indicator plates. Transposase is supplied in trans from a plasmid expression vector. If the transposon integrates into an expressed gene in the correct reading frame, a lacZ fusion protein is produced. Expression of lacZ in the descendants of the original cell is revealed by the outgrowth of blue papillae on X-gal indicator plates. The assay is further illustrated in Supplementary Figure S1 and described in references (24,25). The bacterial mating out assay was based on (24). We first introduced a chloramphenicol resistant derivative of the conjugative plasmid pOX38 into the papillation reporter strain. Transposition of the lacZ-kan reporter from the chromosome into the plasmid is detected by selecting for kanamycin and chloramphenicol resistant transconjugants after mating with a recipient strain. *E. coli* strains were RC5096 [F^-^ fhuA2 Δ(lacZ)r1 glnV44 e14-(McrA-) trp-31 his-1 rpsL104 xyl-7 mtl-2 metB1 Δ(mcrC-mrr)114::*IS10* argE::Hsmar1-lacZ’-kanR] and RC5097 (= RC5096 pOX38::miniTn*10-*CAT); recipient strain was RC5094 [F^-^ araD139 Δ(argF-lac)U169 rspL150 relA1 flbB5301 fruA25 deoC1 ptsF25 rpoS359::*Tn10*].

HeLa cell assays were as described by (26). The respective reporter and transposase expression plasmids were created by replacing the Sleeping Beauty sequences with Hsmar1 sequences. Transposase expression plasmids for the HeLa assays were pRC1241–1249, which encode the wild-type, W118V, W118R, V119T, E122R, L123S, L123N, W118R/E122R and W118R/L123N.

## RESULTS

### Most WVPHEL mutations produce hyperactive transposases

**Figure 2.**
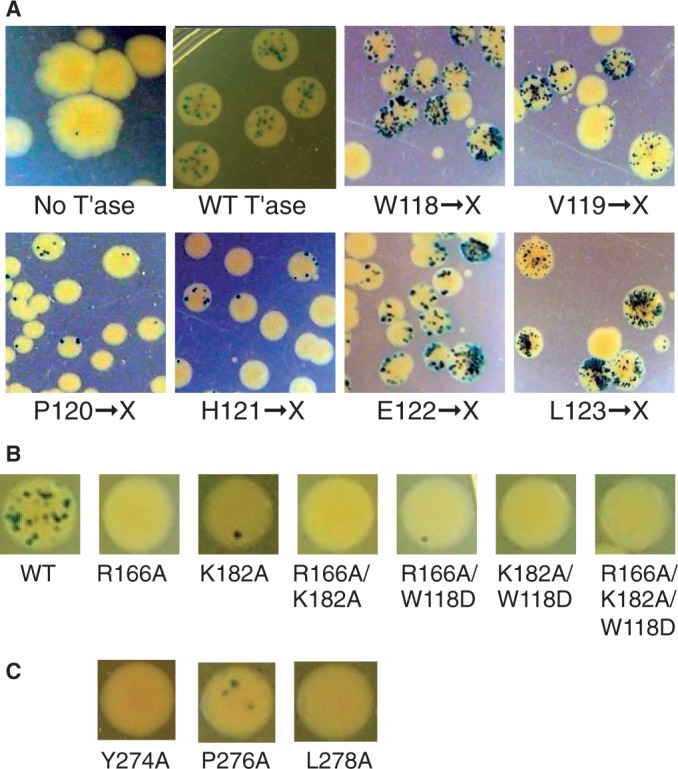
Bacterial papillation assay and genetic screen. An expression vector encoding the transposase was transformed into the papillation reporter strain. Representative fields of papillating colonies are shown. The entire plates are also shown in Supplementary Figure S3. (**A**) Cells were transformed with individual libraries of mutation for each of the WVPHEL residues, as indicated. (**B** and **C**) Site-specific mutations were introduced as indicated. One representative colony is shown.

To provide a visual assessment of the rate of Hsmar1 transposition, we established a papillation assay (illustrated in Supplementary Figure S1). A reporter transposon containing a promoter-less lacZ gene and a kanamycin resistance marker was integrated at a transcriptionally silent locus on the chromosome of a lac*^−^ E. coli* strain. The strain is kanamycin-resistant but because the lacZ gene is not expressed, it produces white colonies on X-gal indicator plates. Transposition yields a lac^+^ phenotype if the reporter integrates into an expressed gene in the correct reading frame. On an indicator plate this is revealed by the outgrowth of blue papillae on the background of a white colony. In the presence of the wild-type transposase a typical colony will develop ∼20 dark-blue papillae after 3–4 days incubation (Figure 2A).

To explore the phenotypes of mutant proteins, we used oligonucleotide-directed mutagenesis to randomize the W118 codon. This yielded a library of plasmids in which all 64 codons are in principle represented equally at this position. DNA sequencing confirmed that all four nucleotides were present at each position (Supplementary Figure S2). When the library was transformed into the papillation reporter strain, most of the papillating colonies did so at a higher rate than wild-type (Figure 2A). There were also a significant number of colonies that did not papillate at all. DNA sequencing revealed that these clones had stop codons or frame shifts introduced during construction of the library. Therefore, it appears that almost all possible substitutions of W118 yield an increase in the rate of transposition.

We generated further libraries of mutations for the remaining five codons of the WVPHEL motif. The V, E and L positions also yielded a high proportion of hyper-papillating clones (Figure 2A). In contrast, mutagenesis of the P and H residues yielded mostly hypoactive clones (Figures 2A and Supplementary Figure S3).

### Interacting-residue substitutions are hypoactive

The W118 residue is sandwiched between a pair of conserved basic residues in the Mos1 clamp-loop region (Figure 1C–E). Therefore, we wondered if a reciprocal mutation would confer a hyperactive phenotype. This was not the case, and the R166A and K182A single and double substitution proteins were hypoactive in the papillation assay (Figure 2B).

The WVPHEL motif also interacts with the conserved YSPDL motif (Figure 1C and D). The most extensive contacts are via the P-H residues, which yielded mostly hypoactive clones in the papillation assay. This suggests that this interface may be fundamentally important over-and-above any regulatory functions of the WVPHEL motif. This seems to be the case because the alanine substitutions of the Y, P and L residues are all hypoactive in the papillation assay (Figure 2C).

### Saturation mutagenesis of the WVPHEL motif

The randomized-codon libraries for the respective WVPHEL residues were individually transformed into *E. coli* and plated for single colonies. We sequenced batches of clones until we had obtained at least 16 of the 19 possible substitutions of the W, V, E and L residues. We assessed the activities of the mutations using a quantitative ‘mating-out’ assay, which measures the rate of transposition of the reporter from its chromosomal locus into a conjugative plasmid (Table 1 and Supplementary Table S1). Of the 17 substitution proteins obtained for the W118 residue, 15 had activities ranging from wild-type to 30-fold more than wild-type. The phenotypes of the V119 substitution proteins were more variable: nine were hypoactive and seven were hyperactive. The E122 and L123 substitutions were similar to those at W118, the great majority being hyperactive.

**Table 1.** Transposition frequencies of individual mutants

Mutation		W	V	P	H	E	L
		Mutant/W.T.	Mutant/W.T.	Mutant/W.T.	Mutant/W.T.	Mutant/W.T.	Mutant/W.T.
Small	G	1	0.03	nd	0.08	0.4	20
	A	8	3	0.5	nd	3	5
Nucleophilic	S	20	7	nd	0.09	6	50
	T	8	7	nd	0.3	8	20
	C	8	2	0.4	nd	1	0.7
Hydrophobic	V	10	1	0.6	nd	10	6
	L	6	0.1	0.4	nd	4	1
	I	4	3	nd	nd	2	3
	M	10	nd	nd	nd	nd	0.8
	P	4	0.02	1	nd	0.2	0.06
Aromatic	F	8	0.2	nd	0.4	2	0.09
	Y	nd	0.06	nd	0.5	5	nd
	W	1	nd	nd	1	2	nd
Acidic	D	0.3	nd	nd	nd	0.08	20
	E	2	3	nd	nd	1	7
Amide	N	nd	0.8	nd	nd	0.6	60
	Q	8	2	0.01	nd	6	3
Basic	H	10	0.3	nd	1	2	0.1
	K	10	0.4	0.04	0.2	7	nd
	R	30	0.02	nd	nd	30	0.9

Transposition frequencies were measured by the bacterial mating-out assay and are the average of three independent experiments. The transposition frequency is obtained by dividing the total number of transconjugants containing the transposon by the total number of transconjugants. Mutant/wild-type transposase (W.T.) figures are rounded to one significant figure. nd, not determined.

To analyze the behavior of the substitutions at the P and H positions, we selected a few clones from the papillation plates across the range of activities observed. The identities of the substitutions were determined by DNA sequencing and their activity quantified in the mating-out assay (Table 1). Only the H121W substitution protein had an activity as high as wild-type. To search for more hyperactive clones, we screened another 1000 clones of the P and H substitution libraries. We sequenced the 16 most active clones but recovered only H121W or wild-type.

Of the 17 mutations obtained for the W118 residue, only the D substitution protein yielded a hypoactive transposase. We hypothesized that it might block the reaction through an ionic interaction with one of the flanking arginine residues (Figure 1C and D). We therefore tested the W118D substitution protein in combination with the R166A and K182A single and double mutants, but none of the combinations rescued the activity (Figure 2B).

### Target site specificity

Previously, a two-hybrid genetic screen suggested that the L124S mutation in Mos1 disrupted subunit interactions (27). In addition, the mutation reduced *in vitro* transposition >20-fold and altered the fidelity of the reaction for the TA dinucleotide target site. In contrast, L123S, the equivalent mutation in Hsmar1, increased transposition by 50-fold in the bacterial assay (Table 1). To assess the target dinucleotide specificity of the Hsmar1 mutants, we sequenced the junctions of four or five independent *in vitro* insertion events for the W118V, V119T, E122T and L123S hyperactive transposases (Supplementary Figure S4). Except for one of the nine insertions produced by the W118 mutants, all were perfectly canonical and duplicated the TA dinucleotide target sequence.

### WVPHEL substitution proteins are hyperactive in HeLa cells

**Figure 3.**
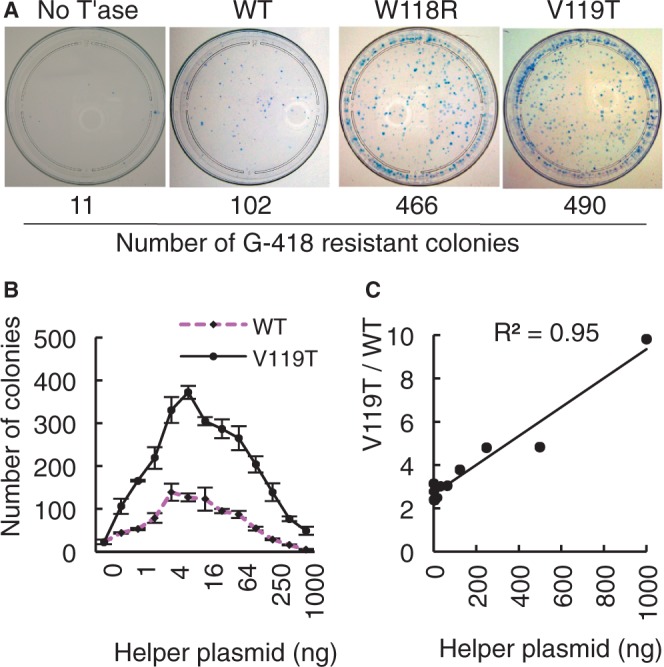
WVPHEL mutants are hyperactive in HeLa cells. (**A**) Cells were transfected with 500 ng donor and 8 ng helper. After 14 days growth in G418 media, the cells were fixed, stained with 1% methylene blue in 70% ethanol and the stable transfectants were counted. Results with additional mutants are presented in Supplementary Figure S4. (**B**) Transfections with 500 ng donor plasmid were titrated with an increasing amount of wild-type and V119T helper plasmids. Error bars are the standard error of the mean after three independent experiments. Transposase expression across this range is not toxic (13). (**C**) The relative activity of the V119T mutation compared with wild-type from part B is plotted against the amount of helper plasmid.

A selection of the hyperactive substitution proteins was assessed in a eukaryotic cell culture assay. The assay is based on the cotransfection of a ‘helper plasmid’ that expresses transposase and a ‘donor plasmid’ carrying a transposon with a neomycin marker (illustrated in Supplementary Figure S5). The rate of transposition is given by the number of stable transfectants obtained after drug selection. Typical results for the W118R and V119T transposases are shown in Figure 3A. Although these substitutions increased activity 30- and 7-fold in the mating-out assay, respectively, they increased activity only 4-fold in HeLa cells. We also tested four other V, E and L substitutions, but in all cases the extent of the hyperactivity in HeLa cells was lower than in the bacterial assay (Supplementary Figure S6). We also tested two double mutants, W118R+E122R and W118R+L123N, which were both less active than either of the single mutants from which they were derived (Supplementary Figure S6).

### WVPHEL substitutions relieve overproduction inhibition

Some eukaryotic DNA transposons are subject to overproduction inhibition (OPI): an increase in transposase concentration beyond a certain point reduces the rate of transposition rather than increasing it. We recreated this effect by titrating the concentration of the wild-type and V119T helper plasmids in the HeLa assay [Figure 3B: for a proof of principle control experiment see Figure 5 and Supplement 1 of reference (13)]. The activity of the wild-type transposase peaked at 4–16 ng of helper and then declined. Peak activity for the V119T transposase was about 3-fold greater, but it was less strongly inhibited at higher expression levels (Figure 3C). At the highest expression level, the advantage enjoyed by the V119T protein had increased from 3-fold to almost 10-fold.

**Figure 4.**
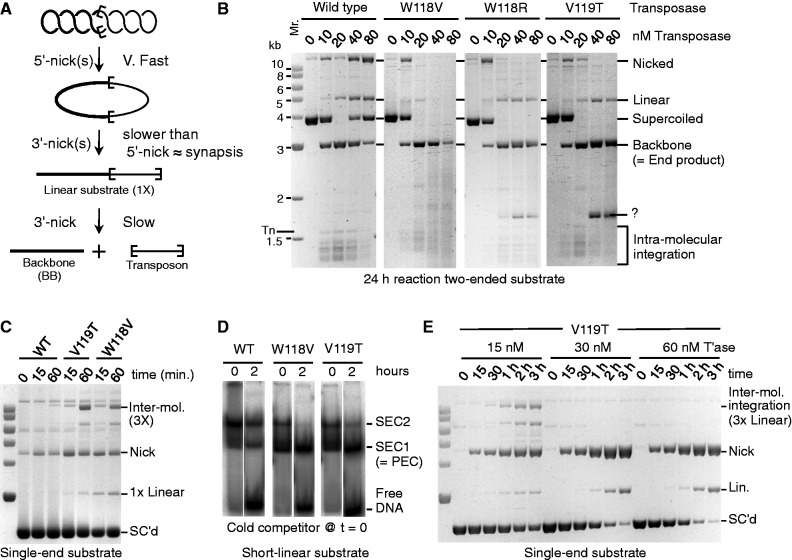
*In vitro* analysis of WVPHEL mutants. (**A**) The excision stage of an *in vitro* transposition reaction with a supercoiled substrate is illustrated. Sequential nicking at the transposon ends yields nicked and linear-substrate intermediates. The plasmid backbone is an end product of the reaction, which provides a direct measure of the efficiency. The excised transposon goes on to yield integration products. Transposon ends are denoted by the open boxes. Products are shown as they appear after deproteination. (**B**) *In vitro* transposition reactions with 6.6 nM double-ended supercoiled-plasmid substrate were titrated with increasing amounts of the indicated transposases. Reactions were deproteinated and analyzed by agarose (1.1%) gel electrophoresis and stained with ethidium bromide. The efficiency of the reaction is given by consumption of the substrate and/or production of the backbone: for details see references (14,23). Tn indicates the position of the excised transposon. However, this is not visible because integration is faster than excision. Question mark indicates a non-canonical product, which had not yet been characterized. (**C**) Kinetic analysis of transposition with 13.2 nM single-ended substrate and 10 nM of the indicated transposases. Reactions were stopped at indicated times and analyzed as in part B. Inter-mol. is the intermolecular transposition product illustrated in Supplementary Figure S8. SC'd indicates supercoiled substrate. Molecular weight markers are as in part B. 1× = 3 kb, 3× = 9 kb. (**D**) EMSA binding reactions contained 2.5 nM radiolabeled transposon end and 5 nM transposase. Complexes were allowed to form for 5 min before addition of a 10-fold molar excess of unlabeled transposon end at time zero. In this figure part, some of the lanes have been stretched vertically to bring the respective bands into alignment and correct for the 2 h difference in loading time. The entirety of the unaltered gels, with additional time points, is provided in Supplementary Figure S10. (**E**) Kinetic analysis of the V119T mutant at the indicated transposase concentrations was as in part C. Molecular weight markers are as in part B.

We next used an *in vitro* assay to assess the sensitivity of the mutants to the excess transposase responsible for the OPI phenomenon. The excision steps of the reaction are illustrated in Figure 4A. Following synapsis, the first nick at one transposon end converts the supercoiled substrate to the nicked intermediate (14,23). Second strand nicking is significantly slower and produces the linear intermediate, followed by the complete separation of the transposon from the backbone. Because we have generated a total of 78 mutant transposases, (Table 1) it would be too time-consuming to characterize them all to the same extent. However, we have performed *in vitro* kinetic analysis with at least two mutations at each of the hyperactive W, V, E and L positions (namely, W118R, W118D, W118V, V119T, V119G, E122T, E122F, L123S and L123G). Their behaviors were consistent with the more detailed *in vitro* analysis presented for selected examples mentioned later in the text.

We began by titrating the supercoiled substrate over a range of transposase concentrations (Figure 4B). The W118V, W118R and V119T mutants continued to excise the transposon at the highest transposase concentration, which was sufficient to inhibit the wild-type protein almost completely (Figure 4B). This confirms the finding from Figure 3B and C suggesting that the V119T mutant is resistant to OPI in the HeLa cell assay, and that this is likely to be a general property of the mutants.

The OPI model predicts that *in vitro* reactions with a relaxed plasmid substrate should be more sensitive to OPI than with a supercoiled substrate (13). This is because the absence of supercoiling reduces the rate of synapsis by lowering the relative concentration of the transposon ends (14). Slow synapsis, in turn, potentiates OPI by providing more opportunity for free transposase dimers to saturate the transposon ends (13). It is worth noting that slow synapsis is the same mechanism that underlies the increased OPI due to the allostery mentioned earlier in the text.

Consistent with the prediction, the reaction with the wild-type transposase was more sensitive to OPI with the relaxed plasmid substrate (Supplementary Figure S7A and D).

This was also true for the W118R and V119T transposases. However, as before, the mutants were less sensitive to OPI than to wild-type and the reactions were faster at all transposase concentrations. This is most easily seen by comparing Supplementary Figure S7D and F. Together these results suggest that the resistance to OPI could in principle be due to faster synapsis with the mutant proteins. Finally, we also note that under OPI conditions the mutant transposase degrade the DNA to various extents (e.g. Figure 4B and Supplementary Figure S7E and F).

### Synapsis and initiation of catalysis

Although a supercoiled inverted-repeat substrate allows all of the chemical steps of the reaction to be observed *in vitro*, synapsis of the transposon ends is too fast to measure accurately. The most stringent conditions for synapsis are when a plasmid substrate encodes a single transposon end. The low relative concentration of transposon ends and their greater freedom of motion mean that synapsis is by far the slowest step and its rate can be estimated from the appearance of integration products (14). Reactions with a single-end substrate yield a complex array of species (illustrated in Supplementary Figure S8), which are resolved into a background smear during electrophoresis. However, the canonical intermolecular product is homogeneous in size and runs in a clear area of the gel. Furthermore, its genesis is unambiguous: synapsis and cleavage of transposon ends followed by integration into an unreacted plasmid yields a linear product three times as large as the substrate (3× in Supplementary Figure S8).

To determine whether the hyperactive transposases perform synapsis faster than wild-type, we analyzed the kinetics of two mutants with the single-end substrate (Figure 4C and Supplementary Figure S9). The W118V and V119T transposases both reacted much faster than wild-type. Because synapsis is the rate limiting step, this shows that the hyperactive transposases are more proficient for this step of the reaction.

As a further confirmation of this result, we assayed assembly of the transpososome using an electrophoretic mobility shift assay (EMSA) (Figure 4D and Supplementary Figure S10). Two complexes are detected in this assay. Single-end complex 2 (SEC2) represents a transposase dimer bound to a transposon end. Single-end complex 1 (SEC1) represents a transposase monomer bound to a transposon end. SEC1 is produced during electrophoresis by the decay of the paired-ends complex and/or an immediate precursor [see Figure 3 and Supplement 2 of reference (13) for a full demonstration and explanation of this phenomenon]. SEC1 can therefore be used as a proxy for the rate of synapsis.

We performed the EMSA assay by mixing transposase with linear transposon ends and allowing 5 min for the complexes to form (Figure 4D). At time 0, we added a 10-fold molar excess of unlabeled transposon end as a competitor. Before addition of the competitor, the mutants had produced much more SEC1 than wild-type. This suggests that they assemble the transpososome more quickly and is consistent with the results in Figure 4C. After addition of the competitor, the labeled transposon end was gradually released as the transposase redistributed onto the unlabeled competitor. This shows that the ‘OFF-rates’ for the mutant and wild-type transposases are approximately the same. This is important because a higher OFF-rate would reduce OPI (13).

When we assayed the hyperactive mutants (W118V and V119T) under OPI conditions, intermolecular transposition was inhibited, as expected (Figure 4E and Supplementary Figure S9A–C). However, nicking and linearization of the substrate increased when the transposase concentration was increased. This contrasts greatly with the wild-type transposase in which these activities are almost completely absent (Supplementary Figure S9A). To characterize the non-canonical nuclease activities of the mutants, we purified the linear product and used restriction endonuclease digestion to show that it arises from double strand cleavage of the DNA at the transposon end.

When we incubated the mutant transposases with a plasmid lacking a transposon end, we found that nicking activity was greatly reduced and the linearization activity was almost completely absent (Supplementary Figure S9D–F). Because these non-canonical activities are largely dependent on the presence of a transposon end, it seems likely that they are due to the decoupling of catalysis from synapsis.

## DISCUSSION

Kinetic analysis of the hyperactive mutants suggests that they are less sensitive to OPI because they assemble the transpososome more quickly. The strongest evidence is provided by reactions with the single-end plasmid, which have the most stringent conditions for synapsis and in which the genesis of the intermolecular product is unambiguous (Figure 4C).

**Figure 5.**
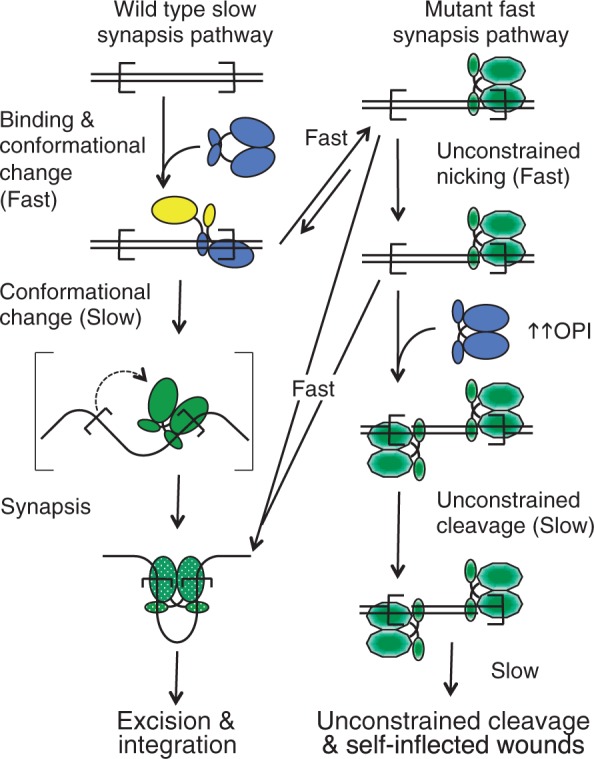
A model for the mechanism of the hyperactive mutants. The transposase DNA binding and catalytic domains are represented as small and large blobs, respectively. Transposon ends are indicated as open boxes. We assume that the free transposase dimer (blue) must have a 2-fold symmetry because this is the lowest energy state for a homodimer. This symmetry is necessarily lost when the dimer binds the first transposon end and then restored when it binds the second transposon end. Conformational changes are indicated by changing colors. Green stippled oval represents the activated catalytic domain. Green hexagon represents the pseudo-activated catalytic domain of the mutants. Full details of the model are provided in the Discussion section.

The behavior of the mutants is most easily appreciated in the context of the normal transposition reaction, which is illustrated on the left side of Figure 5. The first step of the reaction is when a transposase dimer binds to a transposon end. We assume that the free transposase dimer (blue) must have a 2-fold symmetry because this is the lowest energy state for a homodimer. This symmetry is necessarily lost when the dimer binds the first transposon end. The loss of symmetry is associated with an allosteric change between the subunits that reduces the affinity of the unbound DNA binding domain for the second transposon end compared to the first (13,14). To be consistent with a neutron diffraction study on this complex (SEC2), this intermediate is illustrated as an extended structure in which the catalytic domains are far apart and are held together by an interaction involving at least one of the two DNA binding domains (28). After synapsis, which is by recruitment of a naked transposon end, the components on either side of the complex are identical. Presumably the structure must now therefore have a 2-fold symmetry in which the transposon ends are bound with equal affinity. This would require another substantial conformational change (bracketed step). Synapsis culminates in the activation of the complex for chemical catalysis (stippled green). The present results suggest that the WVPHEL motif acts as a relay for signals between opposite sides of the transpososome during assembly. These signals have a dual function in downregulating transposition and coordinating the chemical steps of the reaction.

Coordination is important because aberrant events, such as those that might take place at an unsynapsed transposon end, are non-productive and potentially genotoxic. In Tn*5* and Mos1, coordination of catalysis is reflected in the trans-architecture of the post-cleavage intermediates; the transposase subunit bound to one transposon end contributes its active site to the partner end (15,29). Because the unbound Tn*5* transposase is monomeric, the trans-architecture is established during synapsis. In contrast, mariner transposase binds as a dimer and must therefore have a different mechanism to suppress single-end activity. Our results suggest that this comprises of conformational changes, which are transmitted across the complex in response to transposon-end binding. Interactions between the clamp-loop and the WVPHEL linker, which represent a second trans-architectural aspect of the mariner transpososome structure, act as a conduit for the signals (Figure 1C and D).

*In vitro*, with optimum transposase concentrations, the hyperactive WVPHEL mutants react faster than wild-type (Figure 4C and the panels with 10 and 20 nM transposase in Supplementary Figure S7). Under OPI conditions, when the transposon ends are saturated with dimers and there are few naked ends available for synapsis, the wild-type reaction is inhibited (e.g. Figure 4B and Supplementary Figure S7A). Canonical transposition with the mutants is also inhibited. However, the mutants continue to consume the substrate rapidly (Figure 4E: but for a clear example focus on the 1-h time points in Supplementary Figure S7A and C). If this ‘mutant-OPI-activity’ was taking place within a normal synapsis, the reaction would progress with the usual kinetics. However, it does not, and second strand cleavage is slow, as shown by the accumulation of the nicked intermediate and its slow conversion to linear and backbone under OPI conditions. This indicates that the mutants are more proficient for nicking before synapsis than wild-type. This conclusion is supported by the reactions with the single-ended substrate and the no-end control plasmid (Figure 4E and Supplementary Figure S9). With the wild-type and mutant transposases alike, the canonical 3× intermolecular transposition is absent under OPI conditions (Figure 4E and Supplementary Figure S9). However, as before, the hyperactive mutants perform fast nicking and slow linearization by cleavage at unsynapsed transposon ends. This is illustrated on the right of Figure 5 where the mutants attain a pseudo-activated state (green decagon). Thus, under severe OPI conditions the mutants may remain bound to an unsynapsed end long enough to perform second strand cleavage (Figure 5 bottom right). Such unconstrained cleavage is an inherent danger of the preformed transposase dimer and is not peculiar to the mutants. Even with the wild-type transposase, the tell tale linear product accumulates after a long incubation under OPI conditions (Supplementary Figure S7A, rightmost lane).

Because OPI is caused by saturation of the transposon ends with transposase dimers, it can be overcome by faster synapsis (13). This is because fewer windows of opportunity, represented by the occasional dissociation of a transposase dimer, are required to capture a naked end. OPI can also be overcome by increasing the OFF-rate of the transposase dimer for a similar reason (13). However, the EMSA suggests that this is not the case for the mutants, which release the free transposon end at about the same rate as wild-type transposase (Figure 4D and Supplementary Figure S10).

To understand how faster synapsis is achieved in the mutants, it is important to recall that the ASO mechanism is amplified by allosterism, which lowers the affinity of the single-end complex for the second transposon end. This increases the competition for transposase binding sites by orders of magnitude (13). Therefore, the mutants could achieve fast synapsis if they increased the rate of the conformational change that restores the affinity of the complex for the transposon end. This would be the same conformational change that produces the pseudo-activated state responsible for the unconstrained catalysis in the mutants (Figure 5 blue and yellow to green decagon). However, it is worth noting that the model is a simplification and that synapsis probably involves more sub-steps than illustrated.

In the present work, we have focused on the interactions between the clamp-loop and linker highlighted by the crystal structure of the post-cleavage intermediate. However, it seems likely that the extent and nature of the subunit interface(s) change during the progression of the reaction (e.g. Figure 5). Additional regions of the protein may therefore harbor potentially hyperactive mutations.

The most highly active variants of the PiggyBac and Sleeping Beauty transposons were created by combining several independently isolated hyperactive point mutations (6,7). In both cases, the mutations are distributed throughout the respective proteins. This may be owing to the fact that the original isolates were suboptimal variants generated by genetic drift. However, the dose-response curves for the PiggyBac and Sleeping Beauty transposases hint that these elements may, like Hsmar1, be regulated by an ASO mechanism (13). The ASO mechanism could also fit with a wide distribution of hyperactive mutations because it depends on so many aspects of a protein’s behavior. Under OPI conditions the degree of inhibition is dictated by the ON- and OFF-rates for transposon end binding, the stability of the protein, which affects its steady-state concentration, and the subunit interfaces that mediate allosterism or any of the conformational changes as the reaction progresses.

Finally, it is worth noting that the wild-type transposase has the potential for unconstrained catalysis. This may help to explain the so-called self-inflected wounds detected at Mos1 ends in *Drosophila* (30). It has been suggested that the wounds might be an adaptive feature of the reaction, representing a self-destructive form of autoregulation. However, a more likely explanation may be that Mos1 represents a suboptimal transposase variant, which arose as a result of genetic drift. In contrast, the tight coupling between catalysis and synapsis in Hsmar1 is probably an adaptive feature of the reaction, lowering the genetic burden of the transposon on the host.

## SUPPLEMENTARY DATA

Supplementary Data are available at NAR Online.

## FUNDING

The Wellcome Trust (http://www.wellcome.ac.uk/) [093160 to R.C.]. The funders had no role in study design, data collection and analysis, decision to publish or preparation of the manuscript. Funding for open accesscharge: Wellcome Trust.

*Conflict of interest statement*. None declared.

## Supplementary Material

Supplementary Data
