# Supplementary material for: Hyperactive mariner transposons are created by mutations that disrupt allosterism and increase the rate of transposon end synapsis
Source: Nucleic Acids Res. 2013 Dec 5;42(4):2637–45. doi: 10.1093/nar/gkt1218 (PMC3936726; doi:10.1093/nar/gkt1218)
Supplement: Supplementary Data [file supp_42_4_2637__index.html]

Hyperactive mariner transposons are created by mutations that disrupt allosterism and increase the rate of transposon end synapsis — Hyperactive mariner transposons are created by mutations that disrupt allosterism and increase the rate of transposon end synapsis — Supplementary Data 

# Hyperactive mariner transposons are created by mutations that disrupt allosterism and increase the rate of transposon end synapsis

## Supplementary Data

files

**Files in this Data Supplement:**

- Supplementary Data - pdf file
