# Supplementary material for: Hyperactive mariner transposons are created by mutations that disrupt allosterism and increase the rate of transposon end synapsis
Source: Nucleic Acids Res. 2013 Dec 5;42(4):2637–45. doi: 10.1093/nar/gkt1218 (PMC3936726; doi:10.1093/nar/gkt1218)
Supplement: Supplementary Data [file supp_gkt1218_nar-02934-v-2013-File007.pdf]

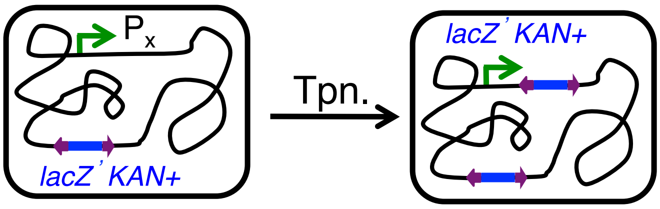

Figure S1 Papillation and mating-out reporter strain

The Hsmar1 transposition reporter, which encodes a promoter-less lacZ gene and a kanamycin resistance marker, is inserted at a transcriptionally silent locus in a lac^- strain of E. coli. Since the lacZ gene is not expressed, the strain produces white colonies on X-gal indicator plates. Transposase is supplied in trans from a plasmid expression vector. If the transposon integrates into an expressed gene in the correct reading frame, a lacZ fusion protein is produced. Expression of lacZ in the descendants of the original cell is revealed by the outgrowth of blue papillae on X-gal indicator plates. Green arrow, promoter; maroon arrows, transposon ends.

For the mating-out assay we introduced a chloramphenicol resistant derivative of the conjugative plasmid pOX38 into the reporter strain. Transposition of the reporter from the chromosome into the plasmid is then detected by selecting for kanamycin and chloramphenicol transconjugants after mating with a recipient strain.

X=any of the twenty amino acids.

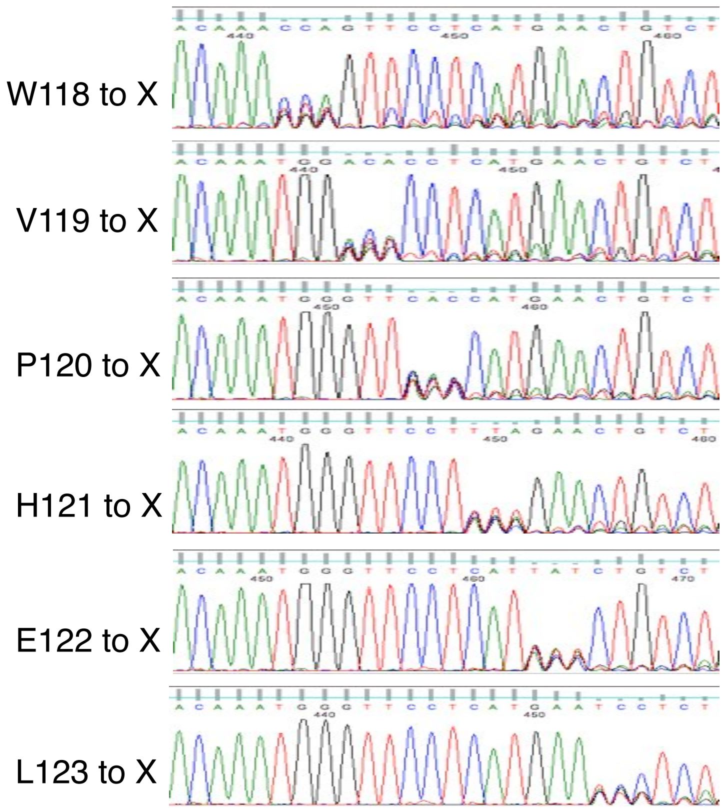

Figure S2 Sequence analysis of randomized codon libraries.

PCR mutagenesis was used to randomize the respective WVPHEL codons in the transposase expression vector pRC880. The respective codons in the mutagenic primer were randomized by using a mixture of all four phosphoramidite precursors. The mutagenized plasmid was transformed into E. coli and plated on selective media. About 6,000 colonies from each mutagenesis were pooled by scraping the plates. Purification of the plasmid from the pooled colonies yielded a library in which the respective codon had been randomized. Successful randomization was confirmed by DNA sequencing of the library pool. As expected, fluorescent signals corresponding to all four bases were present at each of the randomized positions. The mutagenesis procedure introduced a low level of frame shift events in the library, as evidenced by the slight degradation of the fluorescent peaks downstream of the randomized codon.

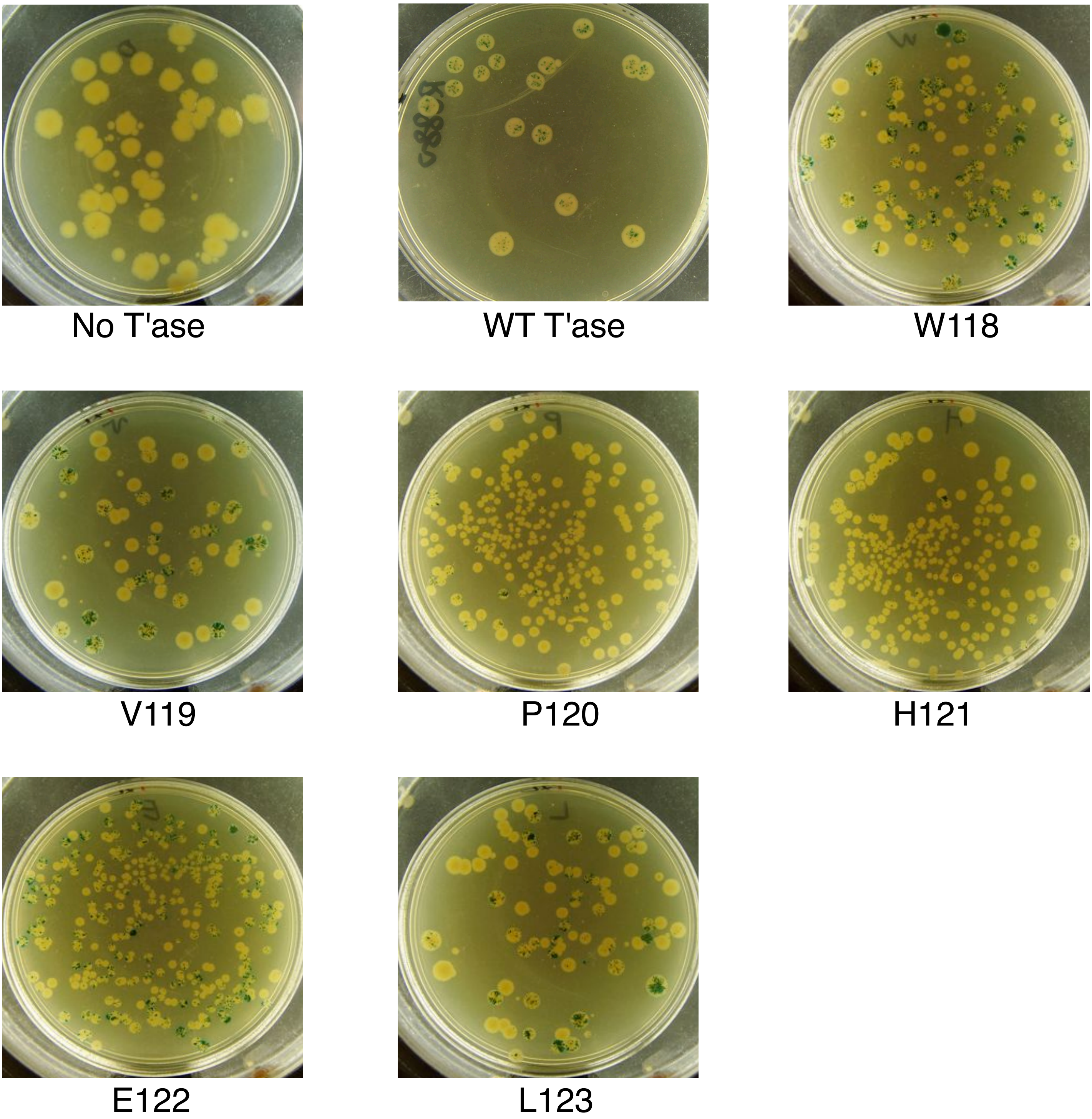

Figure S3 Papillation assay in E. coli

The entirety of the plates from Figure 2B are shown.

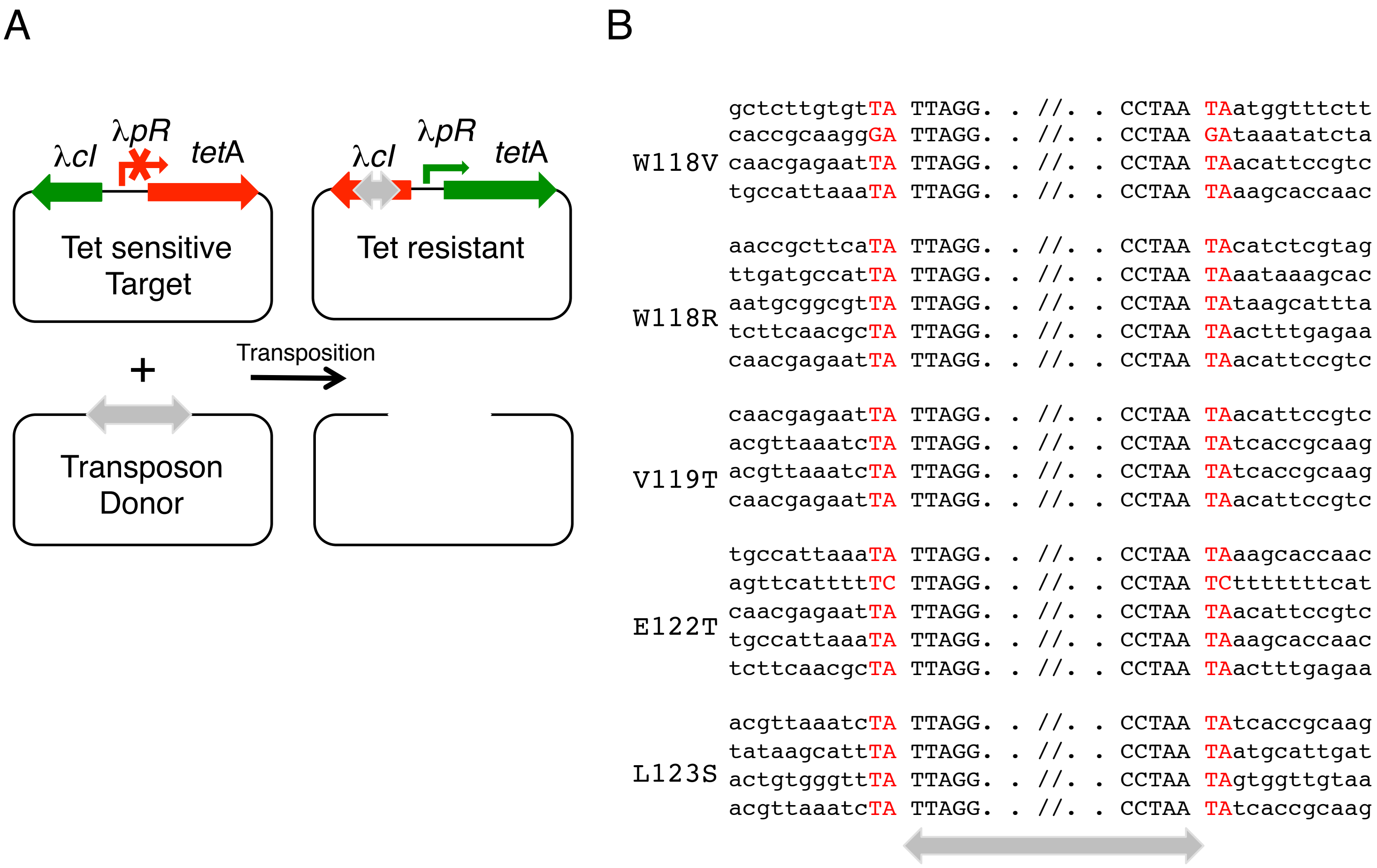

Figure S4 Hyper-active transposase produces canonical integration events.

A, A standard in vitro transposition reaction was performed with the respective hyperactive transposases. A transposon trap plasmid was included in the reaction as a target. The trap consists of a gene conferring tetracycline resistance under the control of the phage λ*pR* operator, which represses transcription in the presence of the λ CI protein. Thick divergent arrows, λ*cI* and *tetA* genes; green fill indicates active states; red fill indicates inactive states; light grey divergent arrows, transposon insertion. Following transformation into E. coli, transposon insertions in the λ*cI* gene are recovered by tetracycline selection.

B, The plasmids form independent tetracycline resistant colonies were recovered. The DNA sequence of the λ*cI* gene was determined and the junctions of the respective transposon insertions were aligned. Except for one example, all were perfect canonical insertions that duplicated the TA dinucleotide target site.

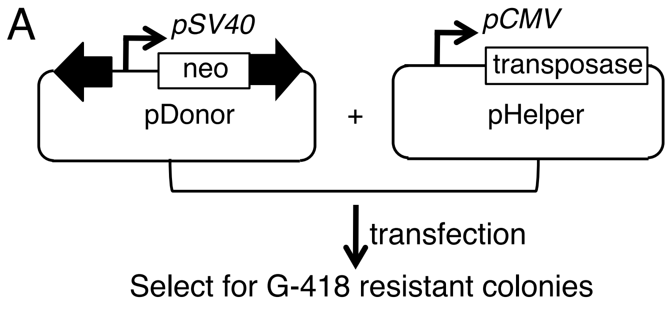

Figure S5 HeLa integration assay

In the HeLa assay cells are transfected with a neomycin reporter plasmid and a helper plasmid expressing transposase. After 24 hours stable transfectants are selected by further growth in G418 media.

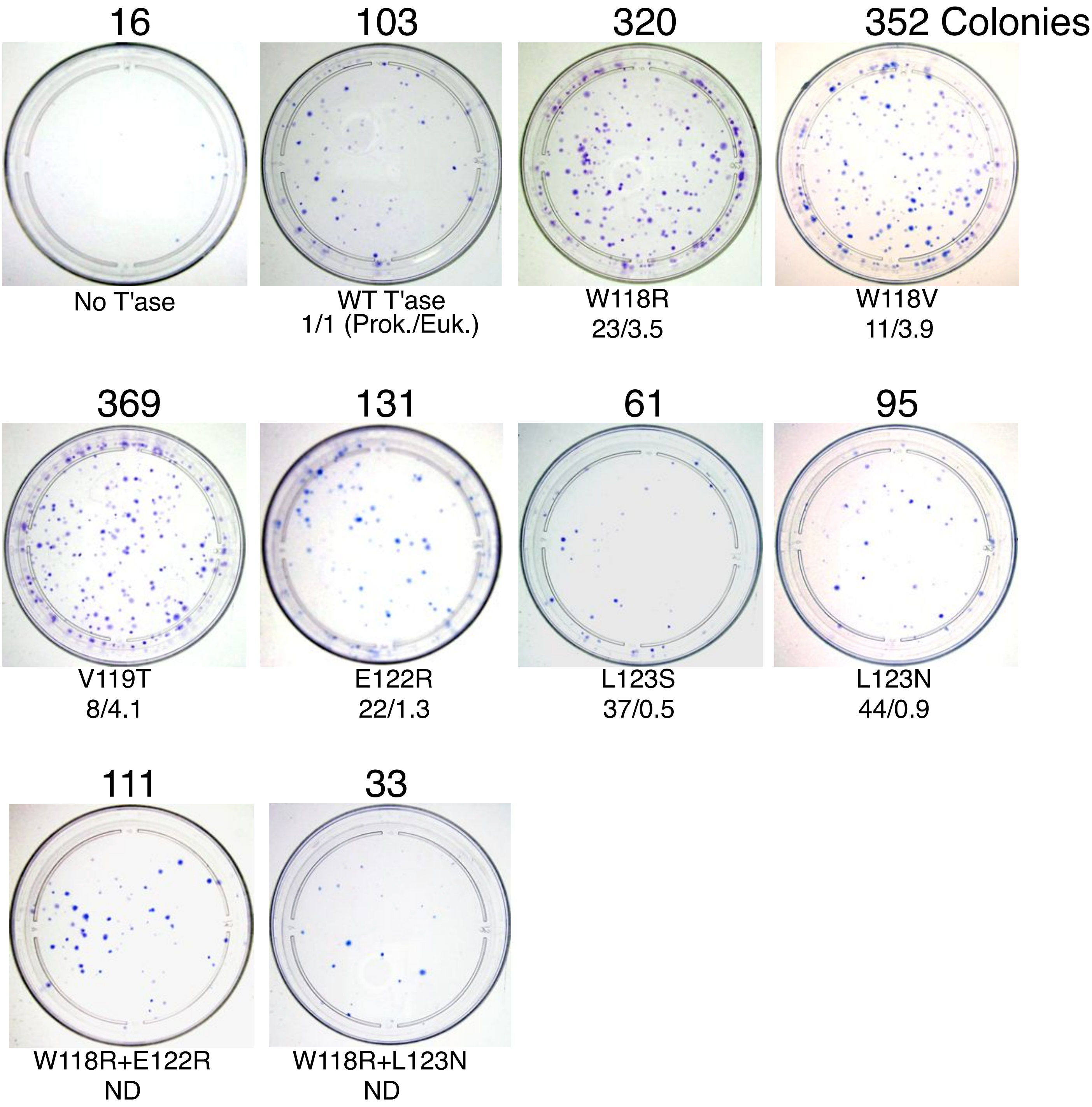

Figure S6 Transposition assay in HeLa cells

A selection of the WVPHEL mutants which were hyper-active in the E. coli papillation assay (Table 1) were assayed in the HeLa cells. The rate of transposition is given by the number of stable transfectants obtained following drug selection. The number of stable transfectants colonies is indicated above each plate. The extent to which each mutant is hyper-active in the bacterial assay versus the HeLa assay is indicated below each plate. ND, not determined.

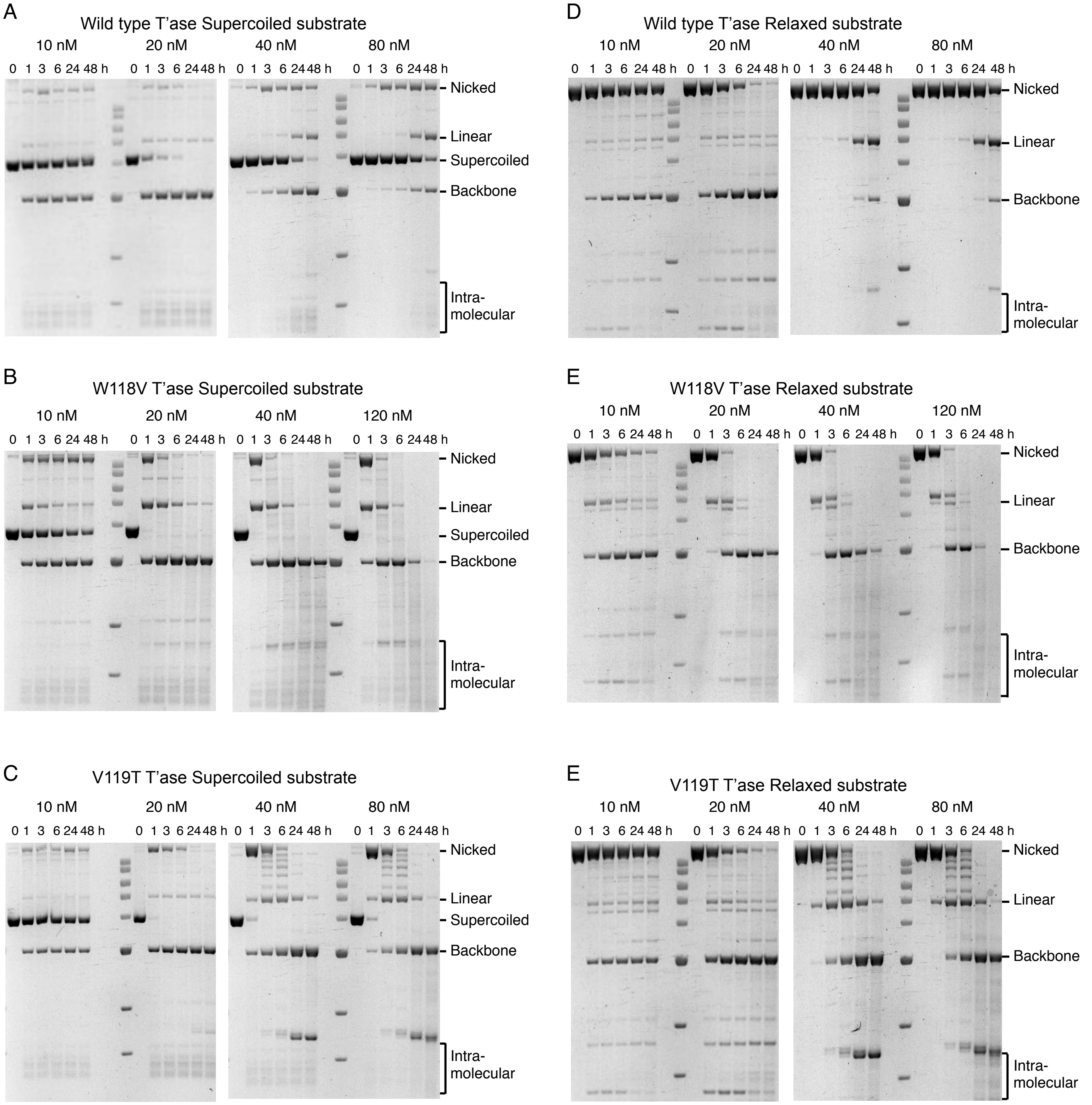

Figure S7 In vitro kinetic analysis of selected mutants with a supercoiled plasmid substrate.

Reactions with various amounts of the transposase mutants were stopped at the indicated times, deproteinated and analyzed by agarose (1.1%) gel electrophoresis and stained with ethidium bromide. The efficiency of the reaction is given by consumption of the substrate and/ or production of the backbone: for details see ref. (14,17). The single- and double-ended plasmids were 13.2 nM and 6.6 nM, respectively. At a given transposase concentration, the ratio of transposase to transposon ends is therefore the same for the single- and double-ended substrates.

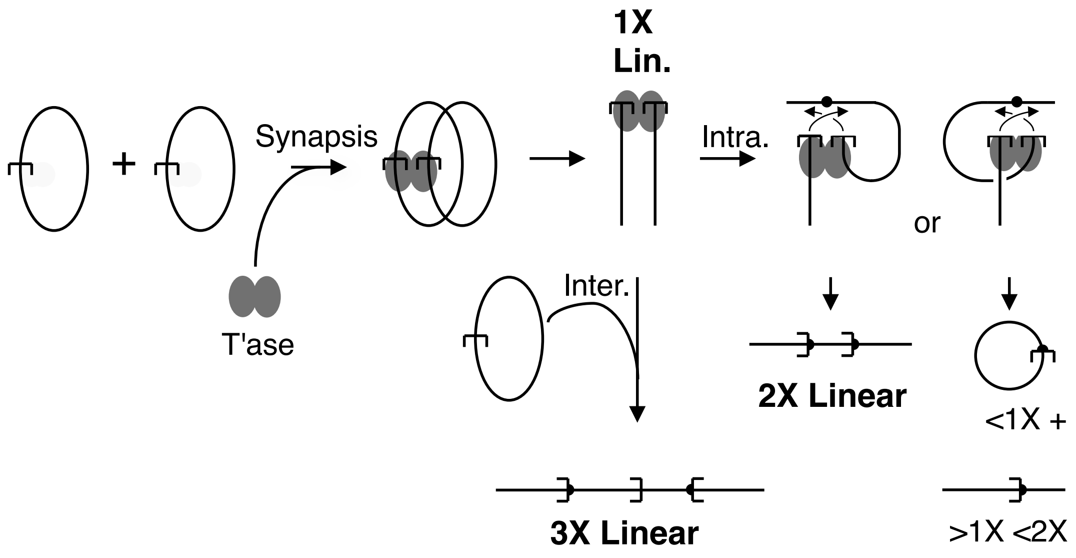

Figure S8 The products of in vitro reactions with single-ends plasmid substrates

Transposon ends are illustrated as open boxes. Supercoiling has been omitted for clarity. The transposase dimer, shaded blobs, interacts with transposon ends on different plasmids. Cleavage yields a linear product the same size as the plasmid (1X). This is the equivalent of an excised transposon in a normal reaction. Integration into an unreacted plasmid yields a product three times the size (3X). Intramolecular integration yields a range of products depending on the location and orientation of the ends with respect to the target site.

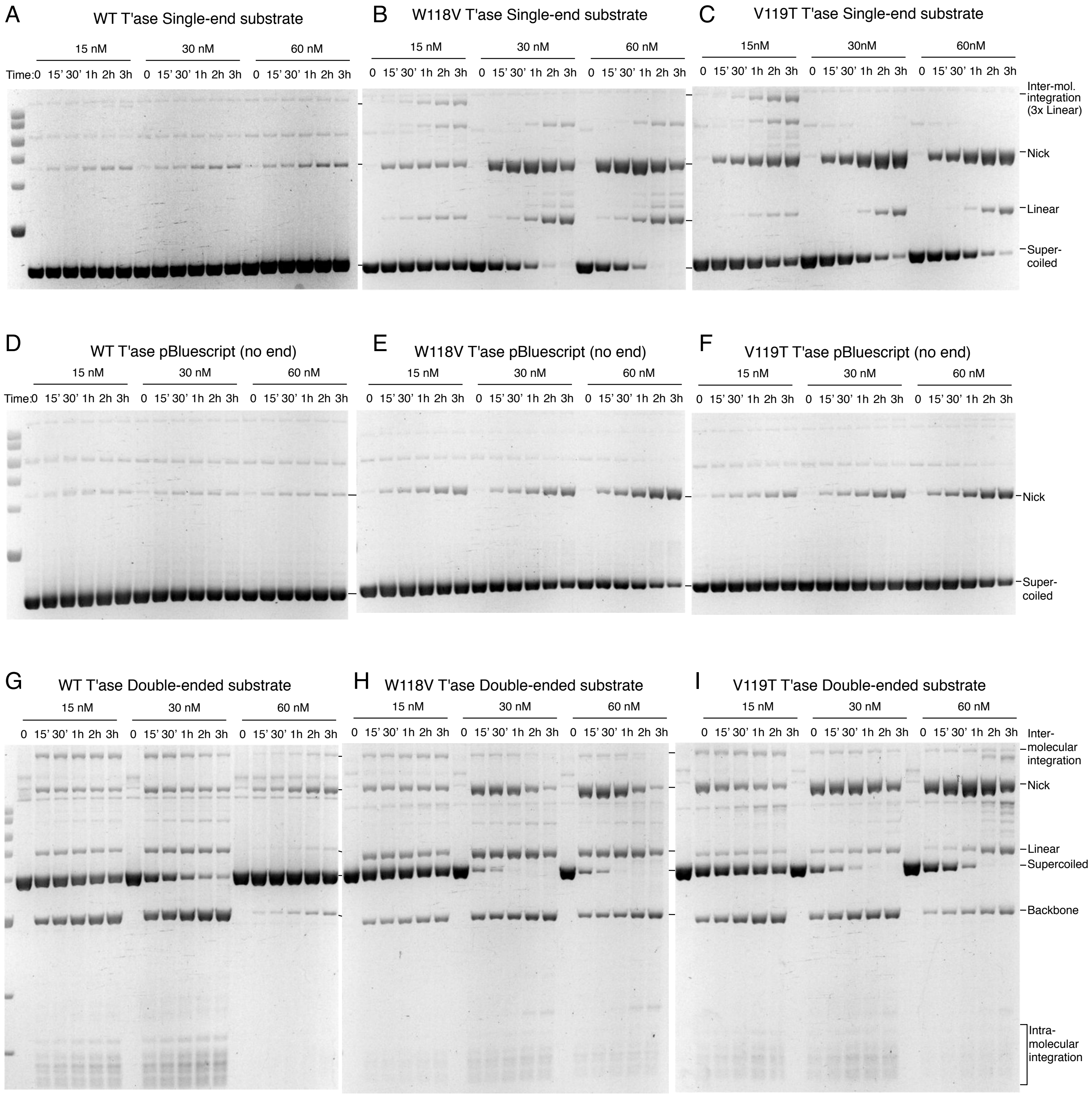

Figure S9 In vitro kinetic analysis of selected mutants with plasmid substrates with and without transposon ends.

Reactions with various amounts of the transposase mutants were stopped at the indicated times, deproteinated and analyzed by agarose (1.1%) gel electrophoresis and stained with ethidium bromide. Further details of the reactions are given in Figure 4 and S8. The single- and double-ended plasmids were 13.2 nM and 6.6 nM, respectively. At a given transposase concentration, the ratio of transposase to transposon ends is therefore the same for the single- and double-ended substrates.

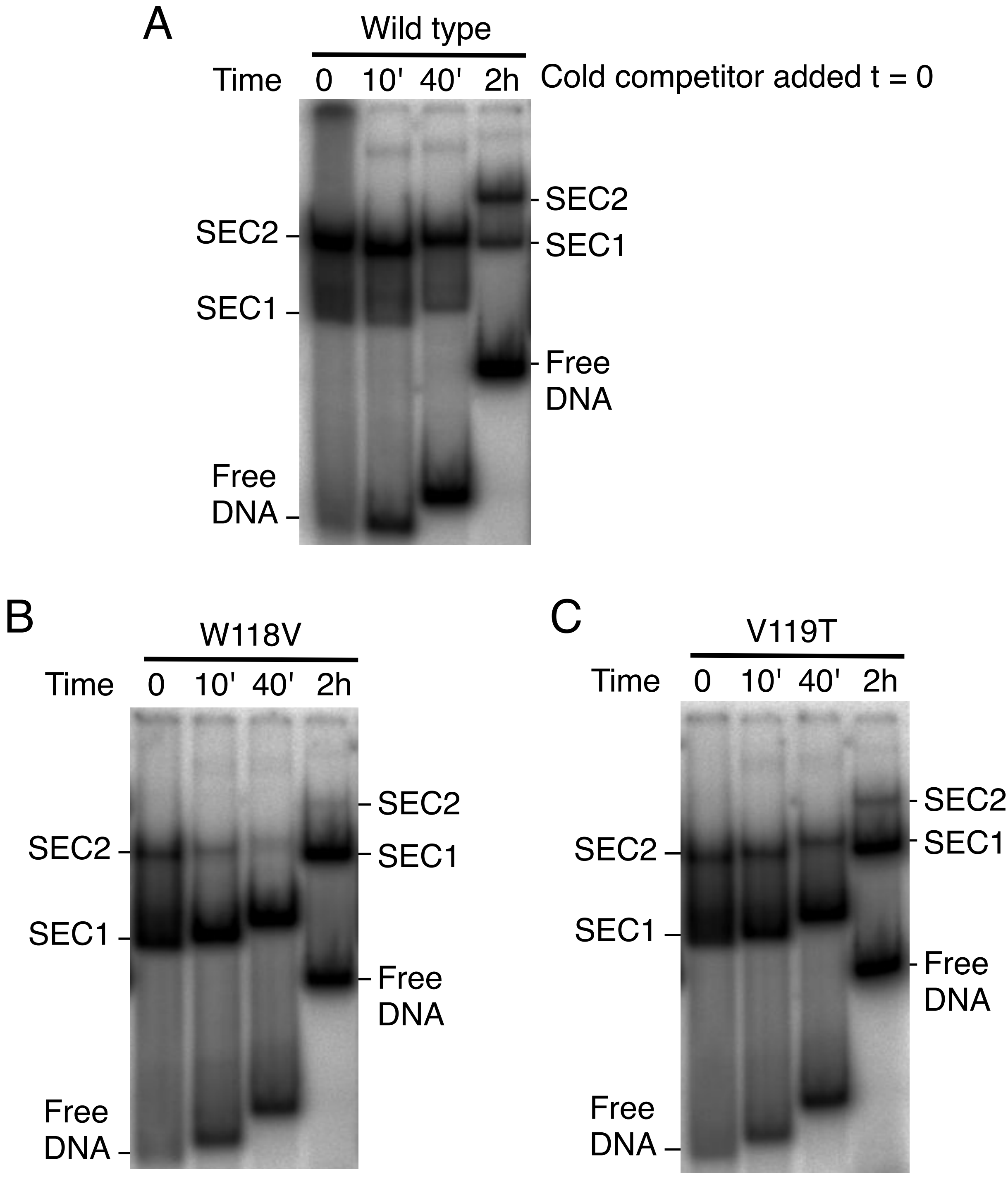

Figure S10 EMSA analysis of transpososome assembly

The gels from Figure 4D are shown in their entirety. EMSA binding reactions contained 2.5 nM radiolabeled transposon end and 5 nM transposase. Complexes were allowed to form for five minutes before addition of a 10-fold molar excess of unlabeled transposon end fragment at time zero. Electrophoresis was started at time zero and aliquots of the respective binding reactions were removed at the indicated times and applied to the gels. As the time course progresses the various bands run progressively further up the gel because of the delay in loading. In Figure 4D the gels have been stretched vertically to correct for this effect and to bring the bands into alignment.

Mutation		W		V		P		H		E		L	
		Transposition frequency	Mutan/ W.T.	Transposition frequency	Mutant/ W.T.	Transposition frequency	Mutant/ W.T.	Transposition frequency	Mutant/ W.T.	Transposition frequency	Mutant/ W.T.	Transposition frequency	Mutant/ W.T.
Small	G	1.6(±0.4) x 10^{-6}	1	3.1(±0.2) x 10^{-8}	0.03	ND	ND	9.2(±0.3) x 10^{-8}	0.08	4.8(±1.3) x 10^{-7}	0.4	1.8(±0.3) x 10^{-5}	20
	A	9.5(±0.9) x 10^{-6}	8	3.5(±1.5) x 10^{-6}	3	5.6(±0.7) x 10^{-7}	0.5	ND	ND	3.7(±0.5) x 10^{-6}	3	6.3(±0.3) x 10^{-6}	5
Nucleophilic	S	2.1(±1.1) x 10^{-5}	20	8.3(±0.4) x 10^{-6}	7	ND	ND	9.4(±2.2) x 10^{-8}	0.09	7.2(±1.4) x 10^{-6}	6	5.6(±0.7) x 10^{-5}	50
	T	1.0(±0.1) x 10^{-5}	8	8.6(±1.1) x 10^{-6}	7	ND	ND	3.8(±0.9) x 10^{-7}	0.3	9.7(±1.3) x 10^{-6}	8	2.4(±0.6) x 10^{-5}	20
	C	1.0(±0.2) x 10^{-5}	8	1.8(±0.2) x 10^{-6}	2	4.7(±1.8) x 10^{-7}	0.4	ND	ND	1.7(±0.5) x 10^{-6}	1	8.6(±0.6) x 10^{-7}	0.7
Hydrophobic	V	1.4(±0.1) x 10^{-5}	10	1.2(±0.2) x 10^{-6}	1	7.7(±0.9) x 10^{-7}	0.6	ND	ND	1.2(±1.6) x 10^{-5}	10	6.7(±2.4) x 10^{-6}	6
	L	6.6(±1.8) x 10^{-6}	6	1.3(±0.9) x 10^{-7}	0.1	4.9(±1.5) x 10^{-7}	0.4	ND	ND	4.8(±1.6) x 10^{-6}	4	1.2(±0.2) x 10^{-6}	1
	I	5.3(±1.2) x 10^{-6}	4	3.0(±0.8) x 10^{-6}	3	ND	ND	ND	ND	2.7(±0.6) x 10^{-6}	2	3.7(±0.9) x 10^{-6}	3
	M	1.2(±0.1) x 10^{-5}	10	ND	ND	ND	ND	ND	ND	ND	ND	9.8(±1.0) x 10^{-7}	0.8
	P	4.9(±1.0) x 10^{-6}	4	2.1(±0.6) x 10^{-8}	0.02	1.2(±0.1) x 10^{-6}	1	ND	ND	2.1(±0.9) x 10^{-7}	0.2	7.6(±0.4) x 10^{-8}	0.06
Aromatic	F	9.1(±0.2) x 10^{-6}	8	2.8(±1.1) x 10^{-7}	0.2	ND	ND	5.2(±1.2) x 10^{-7}	0.4	2.4(±1.2) x 10^{-6}	2	1.1(±0.8) x 10^{-7}	0.09
	Y	ND	ND	7.2(±1.9) x 10^{-8}	0.06	ND	ND	5.5(±2.1) x 10^{-7}	0.5	5.8(±1.2) x 10^{-6}	5	ND	ND
	W	1.2(±0.1) x 10^{-6}	1	ND	ND	ND	ND	1.5(±0.1) x 10^{-6}	1	2.5(±1.2) x 10^{-6}	2	ND	ND
Acidic	D	4.1(±1.5) x 10^{-7}	0.3	ND	ND	ND	ND	ND	ND	9.5(±0.8) x 10^{-8}	0.08	2.1(±1.0) x 10^{-5}	20
	E	2.6(±0.5) x 10^{-6}	2	3.1(±0.8) x 10^{-6}	3	ND	ND	ND	ND	1.2(±0.3) x 10^{-6}	1	7.9(±1.0) x 10^{-6}	7
Amide	N	ND	ND	9.3(±2.0) x 10^{-7}	0.8	ND	ND	ND	ND	7.0(±1.1) x 10^{-7}	0.6	6.6(±0.3) x 10^{-5}	60
	Q	9.0(±1.6) x 10^{-6}	8	2.0(±0.6) x 10^{-6}	2	1.4(±0.1) x 10^{-8}	0.01	ND	ND	6.9(±1.4) x 10^{-6}	6	3.0(±0.4) x 10^{-6}	3
Basic	H	1.4(±0.5) x 10^{-5}	10	3.1(±0.6) x 10^{-7}	0.3	ND	ND	1.2(±0.2) x 10^{-7}	1	2.5(±1.1) x 10^{-6}	2	1.2(±0.2) x 10^{-7}	0.1
	K	1.5(±0.5) x 10^{-5}	10	5.1(±1.5) x 10^{-7}	0.4	4.2(±0.2) x 10^{-8}	0.04	1.8(±1.0) x 10^{-7}	0.2	8.0(±0.1) x 10^{-6}	7	ND	ND
	R	3.0(±0.5) x 10^{-5}	30	2.1(±0.7) x 10^{-8}	0.02	ND	ND	ND	ND	3.6(±0.6) x 10^{-5}	30	1.1(±0.1) x 10^{-6}	0.9

Table 1. Transposition frequencies of individual mutants

Transposition frequencies were measured by the bacterial mating-out assay and are the average of three independent experiments ± standard error of the mean. The transposition frequency is obtained by dividing the total number of trans-conjugants containing the transposon by the total number of trans-conjugants. Mutant/W.T. figures are rounded to one significant figure. ND, not determined.
